# Sexual aggression in the São Paulo nightlife scenarios: a public health concern

**DOI:** 10.1590/S1518-8787.2016050000146

**Published:** 2016-11-29

**Authors:** Zila M Sanchez, Claudia Masur Carlini, Adriana Sanudo, Gabriela Arantes Wagner

**Affiliations:** I Departamento de Medicina Preventiva. Escola Paulista de Medicina. Universidade Federal de São Paulo. São Paulo, SP, Brasil

Brazil withstood a national uproar by a case of mass rape of a 16-year-old girl, broadly addressed on social networks, after leaving a “funk party” on May 25th, 2016. The fact was widely reported on the national and international media^1^ and aroused a motion on social networks with the hashtag *#EstuproNuncaMais* (“No more rape”)^2^.

Between 2013 and 2015, a mixed methods study – which included a cross-sectional survey that interviewed 2,422 people, by systematic sampling, entering or leaving 31 nightclubs in São Paulo selected using a probability proportional to size sampling method^3^, 307 hours of ethnographic observation^4^, eight focus groups with nightclub’ patrons, and 31 in-depth interviews with employees of such clubs – showed these places are a risky environment for sexual abuse. In this scenario, 11.5% (95%CI 7.9–16.2) – weighted prevalence –, of the patrons interviewed at the exit of the nightclubs reported to have suffered sexual aggression, characterized by a perceived sexual abuse that night, from being victim of groping or forced kissing (9.8%; 95%CI 7.2–13.1) to non-consensual sex attempted or executed (1.1%; 95%CI 0.6–2.2). Focus groups statements showed that some nightclubs are more permissive and prone to sexual abuse occurrences, such as funk party nightclubs.

According to a 25-year experienced nightclub manager: “A nightclub is an environment where everything leads to drinking and sex; mainly guys, who believe they can go after girls without ‘excusing’ themselves (…)”. In the terms of a female patron: “Aggression occurs if you say no and the guy grabs you hard by your arm, tries to kiss you without permission, gropes you, and there are some big bullies and if they want something else, they will do it (...)”. The ethnographic observation on funk party nightclubs showed an exclusive presence of sensual dances, and lyrics that stimulate women’s sexual submission, such as “(…) we put it [penis] in her. The gang arrived! The pussy predators!”(…)^5^. However, it should be noted that this type of sexually aggressive behavior does not occur only in funk nightclubs. According to the university students focus group, lyrics of different musical styles stimulate sexual abuse: *“*The lyrics say that the girls will screw around or that men must go around to grope (…) that you have to drink a lot and fuck”.

The definition of a sexual crime in Brazil is “someone who embarrasses another by violence or serious threat, to have sexual intercourse or to perform or allow him to practice other lewd acts”^6^. This document is intended to make the scientific community aware for the need to assist stakeholders in the development of public policies aiming at rape prevention in nightclubs or just after leaving these venues. Considering that our study exposes the occurrence of sexual crimes in nightclubs, it may favor the first step to deconstruct the Brazilian rape culture, by alerting national and international audience about the need of an immediate intervention.

The diagnosis of what occurs in São Paulo’s nightlife is the first step towards preventive actions, based on data from the local reality. Effective preventive approaches to reduce sexual violence in nightlife establishments involve understanding individual behaviors within these places, the nightlife environment structure, and the way behaviors and environment interact to each other.
